# Using qualitative study designs to understand treatment burden and capacity for self-care among patients with HIV/NCD multimorbidity in South Africa: A methods paper

**DOI:** 10.1177/26335565231168041

**Published:** 2023-04-07

**Authors:** Myrna van Pinxteren, Nonzuzo Mbokazi, Katherine Murphy, Frances S Mair, Carl May, Naomi S Levitt

**Affiliations:** 1 Chronic Disease Initiative for Africa, Department of Medicine, 71984 University of Cape Town, South Africa; 2 Department of Health Services Research and Policy, 156606 London School of Hygiene and Tropical Medicine, London, UK; NIHR North Thames Applied Research Collaboration, London, UK; 3 School of Health and Well-Being, College of Medical, Veterinary and Life Sciences, 3526 University of Glasgow, Scotland, UK

**Keywords:** Qualitative research methods, multimorbidity, low-middle income context, treatment burden

## Abstract

**Background:**

Low- and middle-income countries (LMICs), including South Africa, are currently experiencing multiple epidemics: HIV and the rising burden of non-communicable diseases (NCDs), leading to different patterns of multimorbidity (the occurrence of two or more chronic conditions) than experienced in high income settings. These adversely affect health outcomes, increase patients’ perceived burden of treatment, and impact the workload of self-management. This paper outlines the methods used in a qualitative study exploring burden of treatment among people living with HIV/NCD multimorbidity in South Africa.

**Methods:**

We undertook a comparative qualitative study to examine the interaction between individuals’ treatment burden (self-management workload) and their capacity to take on this workload, using the dual lenses of Burden of Treatment Theory (BoTT) and Cumulative Complexity Model (CuCoM) to aid conceptualisation of the data. We interviewed 30 people with multimorbidity and 16 carers in rural Eastern Cape and urban Cape Town between February-April 2021. Data was analysed through framework analysis.

**Findings:**

This paper discusses the methodological procedures considered when conducting qualitative research among people with multimorbidity in low-income settings in South Africa. We highlight the decisions made when developing the research design, recruiting participants, and selecting field-sites. We also explore data analysis processes and reflect on the positionality of the research project and researchers.

**Conclusion:**

This paper illustrates the decision-making processes conducting this qualitative research and may be helpful in informing future research aiming to qualitatively investigate treatment burden among patients in LMICs.

## Introduction

Low and middle income countries (LMICs), including South Africa, are experiencing a rising burden of non-communicable diseases (NCDs), most commonly diabetes, cardio-vascular diseases, hypertension and mental illness.^1,2^ This rising burden is occurring against the background of chronic infectious disease epidemics, most notably HIV and TB.^3^ In the past 15 years, a steep increase in NCDs has been reported in low-income groups in South Africa, who also have the highest burden of HIV.^4-7^ In addition to 8 million people receiving antiretroviral therapy (ART) in South Africa, 4 million people are living with type 2 diabetes and 18 million with hypertension.^1,8,9^ The country is also experiencing an increasing prevalence of cardiovascular disease and an estimated mental illness prevalence of 12%.^10,11^ However, the mental illness prevalence is likely to be an underestimate as it is under-diagnosed in Sub-Saharan Africa.^12^ The convergence of these epidemics is resulting in patterns of multimorbidity which differ from those in high income countries and disproportionately affect socio-economically disadvantaged populations.^13-15^ Multimorbidity increases the complexity of managing diseases, both for the patient and the health services. Consequently, multimorbidity also results in poorer health outcomes, higher health care needs, increased costs and places a greater burden on available health services.^16^

Patients with multiple chronic conditions in low-income settings in South Africa are reliant on a fragmented and overburdened public primary healthcare system. In an effort to improve access to and the quality of care for people with multimorbidity, the National Department of Health has implemented the Integrated Chronic Disease Management model (ICDM).^17^ The ICDM model is currently being implemented in 42 clinics in three of nine South African provinces.^17-19^ Early evaluations indicate that despite efforts made through the ICDM model, chronic care is not well-integrated, staff feel unprepared and there is a lack of tools and equipment to provide adequate preventative care.^3,13^ Patients struggle to adhere to polypharmacy, feel stigmatized and struggle to access services when living far away.^3^

We aimed to examine the impact of multimorbidity on patients’ lives, through the ‘**EX**ploring the **TR**e**A**tment burden and capacity for self-care among patients with HIV/NCD multimorbidity in South Africa to inform the development of interventions’ (EXTRA) study. By conducting in-depth interviews with patients and carers, the EXTRA study aimed to identify, characterize, and understand patient and caregiver workload and capacity associated with self-management amongst people living with HIV/NCDs multimorbidity in rural and urban socio-economically deprived settings in South Africa. It builds on two theoretical models that explore the burden of illness and linked workload and capacity: the Cumulative Complexity Model (CuCoM) and the Burden of Treatment Theory (BoTT), which examine the different self-care practices people with multimorbidity must perform to stay healthy and to respond to the demands of health care providers and the larger health system.^20,21^ Through the EXTRA study, we explore the relevance and applicability of existing theoretical models of NCD treatment workload-capacity from high income countries and adapt them to inform future research and interventions in the LMIC context of SA.

It is within the context of a rising burden of people with multimorbidity and a health system that is aiming to move towards integrated, patient-centred care, that we describe the process of obtaining, analysing, and synthesizing semi-structured qualitative interviews conducted with people with HIV/NCD multimorbidity.^3,4,13,22^ Detailing these processes and methods will allow replication by others aiming to qualitatively investigate treatment burden and capacity among people with multimorbidity in developing countries. This is important, as there is a dearth of research studies on the impact of multimorbidity among patients in South Africa and other LMICs.^7,23^

## Research design

We undertook an in-depth qualitative comparative study in two underprivileged settings in urban and rural South Africa, exploring the interaction between individual patients’ workload and their capacity to take on this workload, using the CuCoM and BoTT. CuCoM examines the interaction between patients’ workload and their capacity to take on this workload.^21,24^ Central to CuCoM is the interaction between *patient workload* as required by the health system and their *capacity* to manage this workload. Capacity includes patients’ physical and mental functioning, socio-economic resources, attitudes and beliefs, and ability to mobilise social support.^21^ BoTT explores the role of the patient and their networks in managing, caring for and supporting the work of being a patient and analyses how patients’ workload is being distributed through and within their networks.^24,25^ Both BoTT and the CuCoM define patient complexity as a dynamic state which considers the personal, social, and clinical areas of patients’ lived experiences as key factors that accumulate over time, leading to complex outcomes.^21,25^ Both the BoTT and CuCoM were designed and tested in the United Kingdom, United States and other high-income settings, and limited research has been conducted to understand treatment burden among chronically ill people in Sub-Saharan Africa.^26,27^ As the EXTRA study intended to explore the relevance and applicability of the workload-capacity models for LMIC-context, both BoTT and CuCoM informed and directed the interview schedule, data collection and analysis of the transcripts.

Drawing comparisons between a South African urban township and a remote rural location allowed us to examine the differences in people’s lived experiences and narratives.^28^ Both settings have varying socio-economic contexts and different access to and quality of health care, suggesting different impacts on decision-making and health outcomes. Focusing on patients’ and caregivers’ lived experiences and those of their caregivers, we explored their accounts of treatment burden. We used the consolidated criteria for reporting qualitative research (COREQ) checklist to report the methods, context, findings, analysis and interpretations of the EXTRA study^29^(Supplement Appendix 1).

### Setting

#### Gugulethu

**Figure 1.**
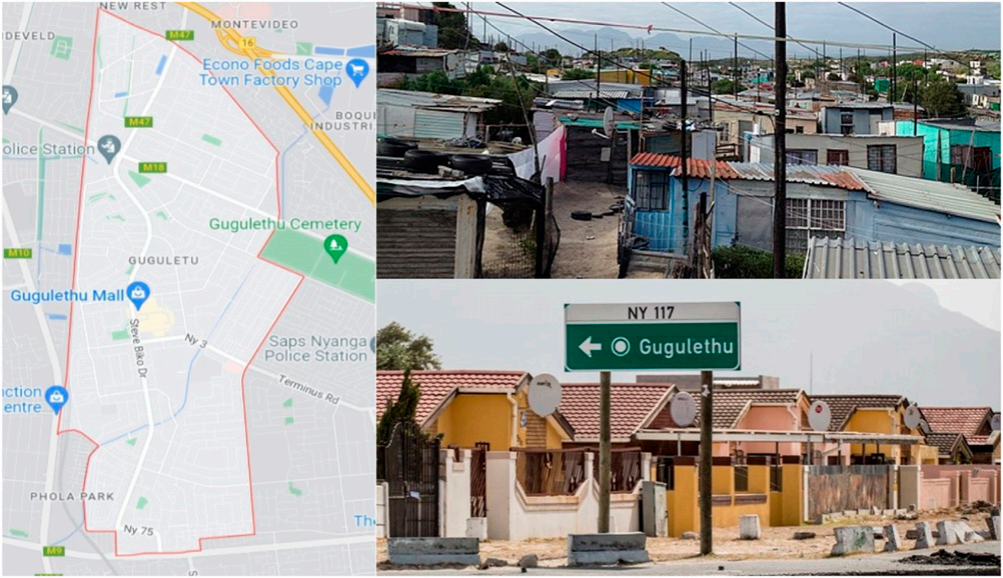
Gugulethu, urban field-site for the EXTRA project.

The urban township of Gugulethu is located 15 km from the centre of Cape Town, in the Western Cape province and was established under the Group Area Act in 1953 as a segregated residential area for black Africans (Figure 1).^30^ It is home to more than 100,000, mostly isiXhosa speaking residents who live in a mixture of formal and informal housing, shacks, and backyard dwellings.^31^ Compared to many other townships, Gugulethu is well-serviced. Schools, shops, and social services are easily accessible. There are three primary care facilities in the area, which offer care for communicable and NCDs, including ART. Additionally, several non-governmental organisations (NGOs) offer health education training and social support programmes. Like the rest of South Africa, the Western Cape province has a quadruple burden of disease, including high prevalence of communicable diseases, NCDs and maternal and child mortality. An estimated 18% of people in the Western Cape live with HIV and 51.6% have hypertension.^32^ Men in the Western Cape also have the highest prevalence (13%) of diabetes in the country,^32^ According to Census 2011, Most people living in Gugulethu are of low socio-economic status, as more than 70% of adults earn a monthly income of R3200 (155 GBP^1^) or less.^33^ Unemployment is high at 39.8% and only 37% of those aged 20 years and older have completed Grade 12 (high school) or higher.^33^

#### Bulungula

**Figure 2.**
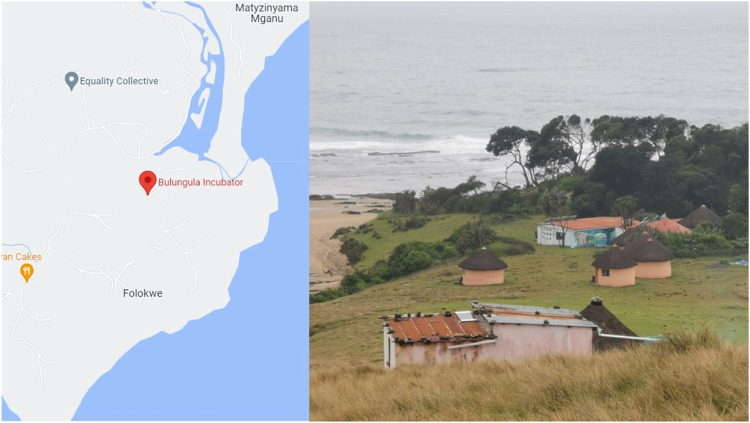
Bulungula, rural field-site for the EXTRA project.

Bulungula is a rural coastal area in the Eastern Cape province (figure 2). The region was part of the former homeland of the Transkei under apartheid, where black Africans retained communal land ownership under the leadership of traditional chiefs.^34^ The villages are sparsely populated, have no tarred roads and the nearest city, Mthatha, is a 2.5-hour journey by car. Most residents have limited access to electricity and running water. People rely on public health services which are far from where they live. For example, some participants had to travel 6-8 hours to get to the nearest clinic. Health status in the Eastern Cape is poor: an estimated 20% of the population has HIV and 49.8% has hypertension.^32^ Women in the Eastern Cape also have the highest prevalence of diabetes in the country (18%).^32^ The rural Eastern Cape is also one of the poorest, most underserviced areas in South Africa and 54% of the adult population is unemployed, relying mostly on grants.^35^ Grants include the government old age pension (R1985 a month, 96 GBP^2^) and the child support grant (R460 a month, 22 GBP^3^).^35,36^

### Sampling

Purposive sampling is widely used in qualitative research, allowing for the identification and selection of cases that are related to the phenomenon of interest.^37^ As experiences can differ by age and sex, we included both females and males with various ages, socio-economic backgrounds, and education levels, we included adults living with HIV and at least one other chronic non communicable disease who attended public primary health care services. Participants could bring their primary carer or supporter if available, but those who came alone were not excluded. All participants had to be 18 years or older and needed to be able to converse in either English, Afrikaans or IsiXhosa, three of eleven official South African languages.

#### Recruitment Gugulethu

In Cape Town, recruitment commenced at the Gugulethu Community Health Clinic (CHC) in late February 2021. A designated fieldworker from the Chronic Disease Initiative for Africa (CDIA) worked closely with researchers to recruit patients. This was a trusted intermediary residing in Gugulethu who was able to use personal contacts and extensive experience as a recruiter to find suitable participants. This was a relatively quick process, as ARV care is offered 5 days a week, is always busy and he was working in Gugulethu CHC for another quantitative multimorbidity study. During recruitment, the fieldworkers introduced the study to possible participants, explained the study objectives and did initial screening before collecting their contact details and setting a date for interviews. In March and early April 2021, the researchers, Nonzuzo Mbokazi (NM) and Myrna van Pinxteren (MvP) travelled to the clinic in Gugulethu three or four times a week to conduct interviews with patients and caregivers.

#### Recruitment Bulungula

Interviews in Bulungula were conducted in April 2021 and participants were recruited from two different villages. In this area, we worked closely with a local NGO, the Bulungula Incubator (BI), that runs different educational and health programmes in the area and assists people in surrounding villages. The BI employs a team of community health workers (CHWs) who conduct house visits, assisting people living with HIV and co-morbidities. We were introduced to CHWs at the BI by a public health specialist who assisted with health programmes run by the BI. Participants received treatment from five different clinics in the district.

### Participants characteristics

A total of 30 people with multimorbidity were included in the study along with 16 caregivers.

#### Gugulethu

In Gugulethu, we invited and recruited 16 people living with HIV and multimorbidity, 9 women and 7 men (See Table 1). In total, 9 participants were unemployed, 4 were retired and 3 were employed or self-employed. The median age of the participants was 56 years, and all had been living with HIV for longer than 5 years. The average education level was grade 10. The most common co-morbidity was hypertension, followed by diabetes. The number of co-morbidities varied from one to five. Additionally, we recruited 10 carers; 5 were participants’ spouses, 3 were family members (son, daughter, cousin) and 2 were friends.

**Table 1.** Characteristics Patients in Gugulethu, Cape Town.

STUDY ID	SEX	AGE	EMPLOYMENT	TIME HIV+	CO-MORBIDITY	CARER PRESENT	MAIN TYPE OF SUPPORT
PU001	Female	60	Unemployed	15 Yrs	Diabetes/Asthma/Hypertension, Heart Condition	Yes	Cousin
PU002	Female	62	Unemployed	21 Yrs	Diabetes/HIV	Yes	Son
PU003	Female	53	Retired	14 Yrs	Stroke/Arthritis/ Asthma/ Depression/Hypertension	Yes	Daughter
PU004	Female	48	Unemployed	17 Yrs	HIV/Hypertension/cellulitis	Yes	Friend
PU005	Female	63	Retired	25 Yrs	HIV/Hypertension/TB in Hip	Yes	Partner
PU006	Female	61	Unemployed	18 Yrs	HIV/Arthritis/Hypertension	Yes	Friend
PU007	Female	48	Unemployed	14 Yrs	Hypertension	No	Husband
PU008	Male	56	Unemployed	19 Yrs	Hypertension	Yes	Friend
PU009	Male	46	Employed	24 Yrs	Diabetes/Hypertension/Depression	No	Wife
PU010	Male	57	Unemployed	19 Yrs	Hypertension	Yes	Partner
PU011	Male	47	Self-employed	5 Yrs	Hypertension	Yes	Partner
PU012	Female	57	Self-employed	18 Yrs	Hypertension	No	None
PU013	Female	65	Retired	17 Yrs	Hypertension/Diabetes/ liver failure	Yes	Partner
PU014	Male	72	Retired	7 Yrs	Hypertension	No	None
PU015	Male	59	Unemployed	5 Yrs	Hypertension/Diabetes	No	None
PU016	Male	46	Unemployed	26 Yrs	Hypertension/Stroke	No	Brother & sister

#### Bulungula

In Bulungula we invited and recruited 14 people living with HIV and multimorbidity, 12 women and 2 men (See Table 2); 11 of the participants were unemployed, 2 were employed and 1 was self-employed. The median age of the participants was 50 years, and all had also lived with HIV for more than 5 years. A total of 7 participants had completed high school (grade 12) and the average grade achieved was grade 10. The most common comorbidity was hypertension and only 2 participants were living with three conditions. We also recruited 6 carers, including 3 daughters, 1 daughter-in-law, 1 mother, and 1 sister wife (a sister through a polygamous marriage).

**Table 2.** Characteristics Participants in Bulungula, Eastern Cape.

STUDY ID	SEX	AGE	EMPLOYMENT	TIME HIV+	CO-MORBIDITY	CARER PRESENT	MAIN TYPE OF SUPPORT
PR001	Female	61	Unemployed	9 Yrs	Diabetes	Yes	Daughter
PR002	Female	59	Unemployed	18 Yrs	Epilepsy	Yes	Daughter-in-law
PR003	Female	72	Retired	15 Yrs	Hypertension	No	None
PR004	Female	60	EPWP^4^	6 Yrs	Heart condition	No	Daughter
PR005	Male	41	Farmer	8 Yrs	Heart disease	No	Wife
PR006	Female	42	EPWP	6 Yrs	Hypertension	Yes	Daughter
PR008	Female	40	Unemployed	9 Yrs	Hypertension	No	Mother
PR009	Female	42	Unemployed	6 Yrs	Hypertension	Yes	Sister-in-law
PR010	Female	63	Unemployed	15 Yrs	Hypertension	No	None
PR011	Female	30	Unemployed	7 Yrs	Hypertension	No	Mother
PR012	Female	48	Unemployed	7 Yrs	Hypertension	No	Sister
PR013	Female	50	Unemployed	11 Yrs	Hypertension	No	Sister
PR014	Male	34	Unemployed	6 Yrs	Hypertension/Stomach condition	Yes	Mother
PR015	Female	63	Unemployed	long time	Hypertension/Cancer	Yes	Daughter

## Data collection

This study used a semi-structured interview guide with open questions, an appropriate method, as there is little qualitative data available on the treatment workload and capacity experienced by people with multimorbidity in rural and urban South Africa.^27,38,39^ The interview schedule was strongly informed by the principles outlined in the BoTT and CuCoM, and an additional analysis of qualitative reviews of patient and care-giver experience of life-limiting chronic conditions.^40,41^ (Supplement Appendix 2, Interview Schedule 1). Questions included focused on both patient and caregiver experiences, expectations and choices about self-care and utilisation of healthcare services. These include socio-economic status; access to healthcare; the quality of health services and interaction with providers; competence and knowledge for self-management, and affective state. In our initial draft of the interview schedule, we included most of the domains proposed by the BoTT and CuCoM and made only minor modifications to the questions to suit our context. However, when reviewing the initial draft of the topic guide in the research team, we realized that the guide was too long and contained too many prompts. In consecutive drafts, we shortened the guide and simplified the prompts for patients. Once in agreement, the interview schedule was piloted by Nonzuzo Mbokazi (NM) (PhD) and Myrna van Pinxteren (MvP) (PhD) with 4 participants and their carers, recruited from Gugulethu CHC. Findings from the pilot were shared with the research team (Katherine Murphy (KM), Naomi Levitt (NL), Carl May (CRM), Frances Mair (FSM)), who provided feedback on the interviews. After the pilot, repetitive questions were removed to avoid duplication and limit the interview time to one hour, to avoid tiring out participants (see appendix 3 + 4, Interview Schedule 2 and 6). We had planned to conduct dyadic interviews, but the pilot revealed that carers preferred to be interviewed separately as they could then speak freely about their experiences of caring for someone with multimorbidity. Subsequently, interviews were conducted consecutively. Despite the experienced challenges, the research team did include the initial 4 interviews in the study, as the responses provided rich interview data. After every interview, fieldnotes were made, containing contextual notes and reflections on the interview- and consent processes. Additionally, the research team participated in monthly online meetings, sharing experiences from interviews, presenting preliminary findings, and discussing strengths and limitations.

In Gugulethu, interviews took place in a consultation room at Gugulethu CHC which offered a quiet and calm space for the participants. For the research team, working in a clinic setting was beneficial, as it allowed us to observe patients’ experiences first-hand, as we saw the long queues outside the clinic gates, stacked folders in the consultation rooms and witnessed organisational clinic processes. Most participants spoke to us immediately after their interactions with health workers and pharmacists, which allowed them to relay recent interactions. In Bulungula, we worked closely with CHWs employed by the BI, and we accompanied them during their home visits. As a result, interviews were conducted in a home environment instead of the clinic, often with family members present.

Interviews with participants lasted between 60-90 minutes in Gugulethu and about 20-30 minutes in Bulungula. This difference in length can be attributed to the fact that urban participants were interviewed in a private room; were keen to talk about the experiences of care they had just received and were generally more knowledgeable about their conditions. In Bulungula, interviews were conducted in a home environment, often with family members walking in and out, which provided less privacy. Additionally, rural participants provided shorter answers when being prompted about receiving and seeking health information, which might be ascribed to the fact that most of them were illiterate and did not have a TV, radio, or smart phone to access health education materials. Despite being shorter, the data from the rural interviews were rich and nuanced and helpful in providing an understanding of patient workload and capacity.

During the data collection process, we followed the COVID-19 regulations as set by the University of Cape Town.^42^ Social distancing and masking rules were observed, and research supplies (pens, money) were sanitized before the start of the interviews. We also insured that the interview location was ventilated by keeping windows and doors open. All those approached agreed to participate, no one declined due to COVID-19 concerns. All interviews were audio-recorded.

### Ethical considerations

This study followed the guidelines from the Principles of Good Clinical Practice and the Declaration of Helsinki.^43^ Ethical approval for this study was obtained from the University of Cape Town (HREC 232/2020) and access to clinics was granted by the Western Cape Department of Health. In the Eastern Cape, we did not recruit respondents directly from the clinics, but received approval from the BI to approach patients identified by the CHWs employed by the BI. Potential participants received detailed information sheets outlining the aims of the study, including contact information prior to providing written informed consent in English or isiXhosa. All participant names and other details that could reveal their identity were removed from the transcribed interviews to ensure anonymity and we assigned study identification numbers to all participants. All project data was stored in a password protected computer which could only be accessed by NM and MvP. Hard copies of consent forms were kept in a locked cabinet at the University of Cape Town accessible by NM and MvP.

## Data Analysis

Before data analysis commenced, all audio-recorded interviews were transcribed and translated into English. We chose to use framework analysis to guide analysis. This is a flexible, but systematic way of structuring, managing and interpreting data that is widely used in the social and health sciences^44-47^ and is suitable when researchers code and analyse data in a team.^44^ For this project, the analysis process was deductive in that we used the BoTT and CuCoM to identify theory-based categories in the data, and inductive, as we sought emergent categories from the data. This was a constant interpretive and reflexive process, divided into the following phases as outlined by Ritchie & Spencer (1994).

### Familiarisation

The researchers (MvP/NM) firstly familiarised themselves with all the interview data by transcribing the audio recordings themselves, translating the Xhosa interviews into English, reading the typed transcripts, and revisiting their field notes.

### Developing a coding framework

Open coding was initially conducted on four transcripts (2 rural and 2 urban) with the assistance of KM and NL. The individually coded transcripts were then reviewed together to establish consistency. After this, a coding framework was developed, and finalised in discussions with the research team (MvP/NM/KM/NL/FSM/CRM) and applied to the remaining transcripts. As analysis proceeded, codes were organised into abstract categories and emerging themes and the framework was continually refined and developed (Supplement Appendix 5, coding framework). Emerging findings were presented during monthly meetings with the research team. This coding framework included categories about relationships and practices through which treatment burden is negotiated and mitigated and focused on the emotional, cognitive, relational, and practical aspects of managing multiple chronic conditions.

### Charting

When all interview transcripts were coded, we further synthesized the data into thematic narrative memos. We selected quotes per category and included contextual notes drawn from our personal reflections and observations of the interviews, our experiences of the field-sites, the organisation of clinic services and the larger socio-economic and historical context of South Africa. Where applicable, different categories were linked together or merged, and comparisons were continually drawn between findings from both urban and rural settings. The memos were discussed with the research team, facilitating the opportunity for members to provide feedback on assumptions and claims made during the data analysis process.

### Further mapping and interpretation

**Figure 3.**
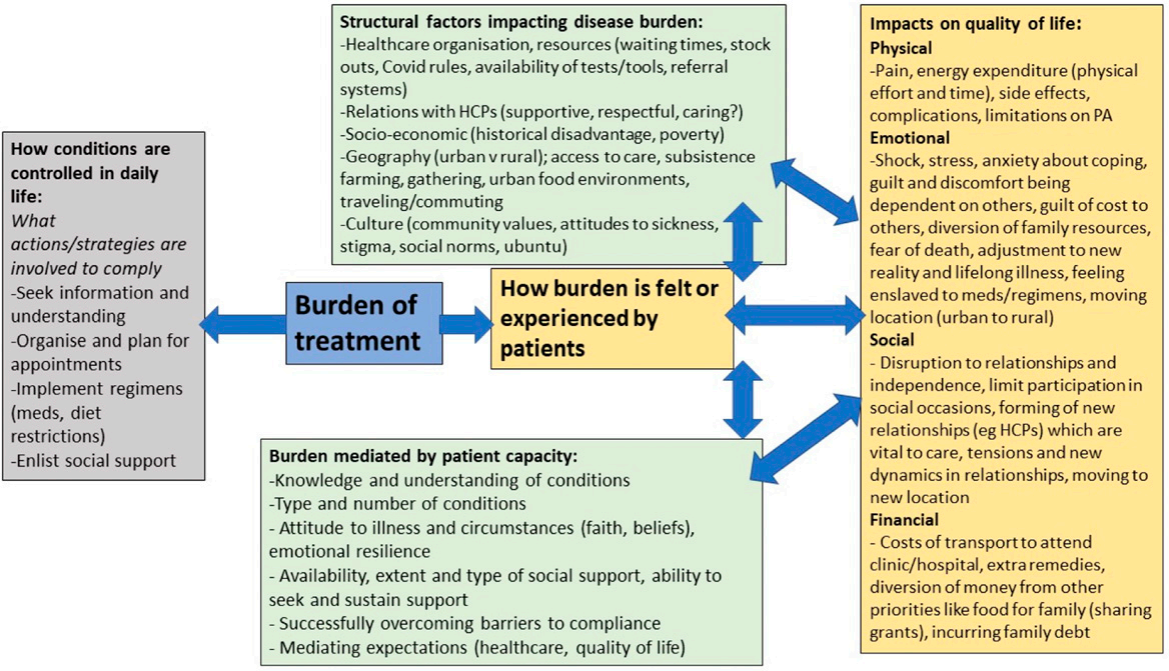
Patient experiences of treatment burden analysis of themes.

The data was further synthesized into various themes as illustrated in figure 3, which also shows the possible mechanisms of interactions between these themes and outlines where findings linked to theoretical concepts. Using this figure, we simplified and unpacked the structural factors impacting patients’ lived experience and their ability to manage their chronic conditions physically, emotionally, socially, and financially. We also illustrated how the burden of treatment is enacted in people’s daily lives and how this is mediated by individual and supporters’ capacity. Two main overarching themes have been written up in detail separately and submitted for publication elsewhere: 1) the impact of precariousness in patients’ treatment workload,^48^ and 2) the importance of social support networks as a mediator of patients’ capacity.^49^ Observations from interviewed carers are to be analysed separately and reported at a later stage. We used Nvivo 12 and Microsoft Excel during the data analysis process.

## Reflexivity and positionality

Conducting research with groups that are considered vulnerable, in this case people living with multimorbidity, requires critical thinking about the positionality of the researcher, the participants, as well as the objectives of the research project. As one of the aims of the EXTRA project was to understand the applicability of the BoTT and CuCoM in low-middle income contexts, the research team consistently reflected on the positionality of the research project and its progenitors and adapted the interview guide to make it suitable for a South African context. The study design and representation of respondents were repeatedly discussed in the research group during the data analysis process. Issues of researcher positionality were also addressed. NM is a black South African woman and MvP is a white European woman, both working as post-doctoral researchers in this project. We also discussed the potential impact of their experiences and positionality during the data collection phase, the analysis meetings and write up of this study. We also worked to enable transferability of findings, as appropriate, by providing further context on the study site in the Methods and Findings section of this paper.

## Discussion

This paper outlines the methodological considerations and strategies considered in a novel qualitative research project aiming to understand the treatment burden and issues affecting capacity to self-care among adults living with multimorbidity in a low-middle income country. We discuss the methodological considerations, impact of reflexivity and positionality on the study design and analysis of the results and highlight the strengths of conducting research in two diverse settings in South Africa.

The findings from the EXTRA study will be presented for discussion with key policymakers and primary healthcare clinicians with the aim of collaboratively formulating a set of practical recommendations to reduce treatment burden and enhance patient capacity in our setting. In this way, the study will help ensure that emic or insider perspectives (patients’ experiences expressed in their own words) is incorporated into the process of primary healthcare reform currently underway in South Africa to integrate and improve the quality of care for patients with chronic conditions and multimorbidity. Results of the study will further be used to critically examine the BoTT and CuCoM, and highlight changes that might be required to make them applicable to and inclusive of patients’ experiences and needs in low-income settings.^50,51^ Incorporating patients’ perspectives into health system improvement interventions enhances the potential that such initiatives are effective, appropriate, and meet the expressed needs of patients.^52^

This study highlights the potential applicability of the BoTT and CuCoM in LMICs when adapted for this context, as both conceptual frameworks prompted participants to reflect on their self-management workload and how the required work impacts their capacity to stay healthy and navigate daily life with multimorbidity.^21,24^ Understanding the methodological implications of using these theoretical models to inform research studies in LMICs is important, as thus far, both BoTT and CuCoM has been applied mainly in high-income contexts and research on multimorbidity is scarce, as only 5% of multimorbidity research globally is currently taking place in LMICs.^53^ The few studies from Argentina, Ghana and Malawi who used BoTT or CuCoM to qualitatively explore self-management among people living with multimorbidity yielded rich results, reporting that patients’ main difficulties relate to managing treatment burden due to financial insecurity, work and caring responsibilities and inadequate health service provision.^26,27,54,55^

The transparency of the research process as detailed in this paper and the use of well-established theories of treatment burden to frame the research and analysis, enhances the potential for replication of this study in other settings. Our successful implementation of the study in two very different settings with different study populations, both which yielded rich information relevant to the research question, also demonstrates its potential transferability or relevance to a wider or alternative setting. The study shows that there are benefits to having qualitative comparison groups; including that comparisons can reveal greater variation in the dimensions of a common experience depending on circumstance, how differences in context yield different outcomes and how different groups can tell similar stories with variations reflecting their particular culture, thus revealing nuances in cultural and social meanings.^56^ In our study, comparisons between urban and rural groups prompted further questioning of the data and a deeper analysis as we sought to understand what lay behind their similarities and differences.

Further strengths of the study include our prolonged engagement in the study settings, which deepened our understanding of the context in which our study population lived. In the urban area, frequent and lengthy visits to the clinic gave us insight into the organisation and running of the health services; and in the rural setting, staying in local accommodation for 2 weeks and gaining access to patients’ homes gave us first-hand experience of our respondents’ way of life. Additionally, the extensive journal notes that we kept throughout the data collection phase and peer de-briefings after each interview all assisted in the critical reflection of our positionality and in checking the validity of our initial understanding of the data. Regular presentations and discussions with the broader research team during the data analysis phase helped us clarify and test our interpretations, check for gaps and biases and apply the relevant theoretical concepts. Data collection included 30 patients and 16 carers, provided rich and nuanced experiences, and data saturation was reached in both settings. Therefore, findings of this study allowed us to develop a robust understanding of the study phenomenon.^57^ Nonetheless our study has several limitations. Firstly, our sampling was constrained in the rural setting by our dependence on the CHWs to identify and access participants. Whilst we intended to sample equal numbers of men and women, we only succeeded in including 2 men in the rural sample due to the migration system in South Africa where many men leave their rural homesteads to seek employment opportunities, returning home only infrequently.^58^ Men also generally have poorer engagement with HIV and NCD care, worse health outcomes and higher mortality than women.^59–62^ This was apparent in our study, as five female participants in Bulungula were widows, who lost their partners due to late presentation to services and complications of HIV/AIDS. Secondly, all recruited participants lived with HIV/NCD multimorbidity which excluded patients with different patterns of multimorbidity who might have different experiences. These experiences would be worth exploring in future research. Only two of our participants had self-reported depression. Therefore, this study does not provide significant insights into additional issues that might be faced by people with mixed mental health and physical multimorbidity, which are currently significantly underreported in this context.^63^ Thirdly, the study focused on isiXhosa speaking South Africans, which leaves out many other cultural groups, who might view the topic through different cultural constructs and have different social support structures to draw on in managing their conditions.

### Recommendations

For similar, comparative studies, researchers should carefully consider the field-sites for data collection as contexts can differ within the same country or province. Working with experienced fieldworkers or gatekeepers is advantageous as they can facilitate recruitment of diverse participants, streamlining the recruitment process. Furthermore, we recommend researchers to design the data analysis procedures before fieldwork commences, so considerable time can be allocated to this part of the study. Sharing narrative memo’s and organising discussions within the research team to discuss findings further aids data analysis and facilitates interpretation and contextualisation of the research data.

## Conclusion

This paper reflected on the process of obtaining, analysing, and synthesizing semi-structured qualitative interviews conducted with people with multimorbidity residing in low-income settings in rural and urban South Africa. Sharing narrative memo’s in meetings with the research team to discuss findings is crucial for facilitating interpretation and contextualisation of data, and obtaining different perspectives. Using BoTT and CuCoM as theoretical frameworks further facilitates the examination of broader issues that impact health and care including poverty, living conditions and social disadvantages which all affect individuals’ capacity to manage their treatment burden.^7^

## Supplemental Material

Supplemental Material - Using qualitative study designs to understand treatment burden and capacity for self-care among patients with HIV/NCD multimorbidity in South Africa: A methods paper Click here for additional data file. Supplemental Material for Using qualitative study designs to understand treatment burden and capacity for self-care among patients with HIV/NCD multimorbidity in South Africa: A methods paper by Myrna van Pinxteren, Nonzuzo Mbokazi, Katherine Murphy, Frances S Mair, Carl May and Naomi S Levitt in Journal of Multimorbidity and Comorbidity

Supplemental Material - Using qualitative study designs to understand treatment burden and capacity for self-care among patients with HIV/NCD multimorbidity in South Africa: A methods paper Click here for additional data file. Supplemental Material for Using qualitative study designs to understand treatment burden and capacity for self-care among patients with HIV/NCD multimorbidity in South Africa: A methods paper by Myrna van Pinxteren, Nonzuzo Mbokazi, Katherine Murphy, Frances S Mair, Carl May and Naomi S Levitt in Journal of Multimorbidity and Comorbidity

Supplemental Material - Using qualitative study designs to understand treatment burden and capacity for self-care among patients with HIV/NCD multimorbidity in South Africa: A methods paper Click here for additional data file. Supplemental Material for Using qualitative study designs to understand treatment burden and capacity for self-care among patients with HIV/NCD multimorbidity in South Africa: A methods paper by Myrna van Pinxteren, Nonzuzo Mbokazi, Katherine Murphy, Frances S Mair, Carl May and Naomi S Levitt in Journal of Multimorbidity and Comorbidity

## Notes

1. Calculated on 10/01/2023 (3,200 ZAR to GBP - South African Rand to British Pounds Exchange Rate (xe.com)).

2. Calculated on 10/01/2023 (1,985 ZAR to GBP - South African Rand to British Pounds Exchange Rate (xe.com)).

3. Calculated on 10/01/2023 (460 ZAR to GBP - South African Rand to British Pounds Exchange Rate (xe.com)).

4. The Expended Public Works Programme (EPWP) is a government funded programme aimed to relief poverty by providing temporary work for the unemployed (Expanded Public Works Programme | South African Government (www.gov.za)).
