# Supplementary material for: Using qualitative study designs to understand treatment burden and capacity for self-care among patients with HIV/NCD multimorbidity in South Africa: A methods paper
Source: J Multimorb Comorb. 2023 Apr 7;13:26335565231168041. doi: 10.1177/26335565231168041 (PMC10088413; doi:10.1177/26335565231168041)
Supplement: Supplemental Material - Using qualitative study designs to understand treatment burden and capacity for self-care among patients with HIV/NCD multimorbidity in South Africa: A methods paper [file sj-pdf-2-cob-10.1177_26335565231168041.pdf]

Interview Schedule Draft 1.0

Finding	Measurable construct	EXPERTS I factors that shape patient experience of long-term conditions	Research questions (participants)	Interview questions
1. **Structural, spatial and systemic disadvantages are important factors that inhibit active engagement with formal** [illegible]	**Socio-economic status**	Patient experience is negatively effected by inequalities related to income (1, 2); age and gender (3-6); and ethnicity (7).	How are participants' experiences of health and healthcare shaped by inequalities related to income, age and gender; and ethnicity? How do they define and experience inequalities? Can they afford the cadrugs, aids, and services that they need? What work do they need to do to overcome these problems?	How do you think your age/gender/income affects your health (physical; mental) and your experience of health care?<br><br>*Prompts:* What are the costs associated with caring for your health? Are there things that you need to take care of your health that you cannot afford (meds; food; aids; services)? How does your age affect your access to health services or the way HCPs treat you or talk to you? Is there anything that you do to try and overcome such difficulties?<br><br>*For the carer:* What are your thought about these issues?

	**Spatial location**	Patients and caregiver experience is negatively affected by unequal access to services and transport (8-11), and unequal distribution of environmental problems (8-11).	How are participants' experiences of health and healthcare shaped by the natural and built environment (housing quality, availability of utilities, security and safety)? How are they shaped by distances needed to travel to receive care, the availability and costs of transport? What work do they need to do to overcome these?	What are some of the difficulties you experience in getting to health services (clinic; hospital) and getting the care you need?<br><br>*__Prompts:__*<br>Time; work and family commitments; transport.<br>How do you think the area in which you live affects your ability to manage your health well (crime; diet; physical activity; availability of community resources)?<br><br>*__For the carer:__* What are the difficulties you, as the carer, experience in helping the pt access services?

	**System quality**	Patients and caregiver experience is negatively affected by poor professional support and material assistance (10, 11), continuity of care (9); coordination of services (12-15) and intra-professional communications (11-14, 16). Limited professional expertise in multimorbidities (17), and slow professional responses to anxieties and emergencies (9) are also markers of poor system quality.	How are participants' experiences of health and healthcare shaped by interactions with healthcare providers and professionals? What work do participants have to do to engage with them and manage their own care processes? Are there specific system or professional probelems that participants need to overcome? What work for them or others does this call for?	What is your experience of the health services you use? What is good/not so good? What would you like to see improved/changed?<br><br><u>*Prompts:*</u><br>*HC environment:* What is the quality of the facilities (cleanliness; security; comfort; toilets)?<br>*Organisational:* What do you think of the way the health services are organized (opening and closing times; waiting times; queue management; co-ordination and continuity of care; role of different *HCPs;* location of pharmacy; referral; pt records)?<br>*Clinical staff:* What is your experience of the drs and nurses (perceived expertise/clinical skills; professionalism; attitude to pts - way they treat and talk to pts)?<br>*Other staff* (security; admin): how professional and efficient are other staff at the clinic? How do they treat pts?<br><br><u>*For the carer:*</u> Do you have anything to add?

**2. Patients and caregivers experience multiple affective, cognitive and interactional disadvantages as they seek to participate in encounters with clinicians, decisions about their formal**	**Cognitive advantage**	Patients and caregivers are cognitively disadvantaged by lack of educational resources (18, 19) and information (20, 21). Disadvantage is exacerbated by poor understanding of disease and disease progression (3, 5, 9, 11, 12, 14, 15, 22-27) (17, 23, 27, 28). These may lead to hypervigilance about symptoms, poor or confused symptom recognition (5, 12, 15, 18, 28), and ill-preparedness for crises (12).	What do participants need to do to find out about their health problems? Who do they need to engage with to do this, and what work does this call on them to do? How do they personalize this knowledge to their own circumstances?<br><br>What do participants do to understand and prepare for symptoms and their exacerbation, what work is involved in this, what support is available?	What do you understand about your conditions? How have you gained info/knowledge about your two conditions and how to take care of your health?<br><br>*<u>Prompts:</u>*<br>Best sources of info; level of trust of available info and sources; relevance to your personal circumstances? What do you think you know enough about? And what would you like to know more about?<br><br>When you experience symptoms that concern you and you need to understand more about them, where do you go to get information and support?<br><br>*<u>For the carer:</u>* How have you learnt about these conditions and how to help patients manage them? How well informed do you think you are? Have you had access to any resources which specifically address your needs as a carer? If so, please describe.

	**Affective state**	Patients and caregivers may experience changes in self-identity, along with reduced self-esteem and self-worth, and loss of social functioning (3, 4, 15, 27, 29-32). They may experience increased fear, anxiety, isolation and discomfort (10, 18, 31, 33-35), and this may lead to inappropriate responses to acute episodes of illness (2, 7, 16, 18, 36, 37).	How do participants experience and manage issues connected with stigma, self-esteem, and social functioning? What problems do they face? What do they need to do to achieve this?	How has having HIV/T2D/Dep affected you emotionally? How did getting another chronic condition affect you emotionally/psychologically? Who can you depend on for emotional support? Has having HIV/T2D/Dep affected your personal relationships in any way? Has it affected the way you see yourself or think about yourself?<br><br>*For the carer:* Do you have anything you would like to add from your perspective? How have you coped emotionally as the pt's main carer?
	**Interaction quality**	Good professional-patient relations (7, 9, 16), and individualized timing and type of information delivery (19, 20, 38, 39) can have a positive effect on patient and caregiver experience. Fear of death (3, 15, 40-42) negatively affects patient-professional interaction.	What do participants do to manage and improve the quality of their relationships with health providers and professionals? Do they have particular interaction strategies that they pursue to personal objectives? What do they need to do to achieve these?	How would you describe your relationship with HCPs (drs and nurses)? How do they help you?<br><br>*Prompts:*<br>How do you feel when interacting with them? How are you in the interaction? How much do you feel part of decision making about how to manage your health? What do you think would improve the quality of your interaction with your HCPs? Does having two chronic conditions change anything in your relationship with HCPs?<br><br>*For the carer:* What is your experience of interacting with the HCP when you accompany the pt?

**3. Patients and caregivers value resilience, functional performance and social support that make a practical contribution to formal healthcare and self-management.**	**Adaptation to disruption**	Patient and caregiver experience is positively affected by adaptive processes (15, 43, 44); the normalization of experienced symptoms and physical limitations (2, 39, 40) and normalization of self-management strategies (43). Patient and caregiver resilience (8, 24, 29, 44), capacity to manage uncertainty (6, 7, 9, 34), tolerance of disruption of everyday activities and competing clinical priorities (12, 15, 17, 24, 42, 45) also positively affect patients experience.	What do participants do to adapt to thir health and healthcare problems? How do they experience physiological and psychological effects and limitations and what self-manaagement strategies do they use (if any)? How do they 'smooth out' the disruptive effects iof health and healthcare problems?	How has having both HIV/T2D/Dep changed your life (physical & psychological effects; limitations on way of life, work and routines; impact on relationships; family life)?<br><br>*<u>Prompts:</u>*<br>How was it to have a second chronic condition to cope with?<br>How have you coped with these changes?<br>What has helped you adapt to living with two chronic conditions?<br>How easy or difficult has it been to accept our situation? And how have you overcome some of the difficulties?<br>How do you manage work in your situation (time off for appointments and illness; attitude of co-workers and boss; limitations on ambitions?)<br><br>*<u>For the carer:</u>* How has your caring role affected or changed your life (eg: limited your activities; changed your routine and relationships)?<br>How have you adapted to these changes?

	**Caregiver support**	Caregiver support is defined by material assistance (2, 29, 45, 46), symptom management and self-management (18, 41, 44, 47) and emotional and relational solidarity (16). It is negatively affected by perceived burdens and workload that interfere with normal life (6, 9, 21, 32, 46, 48, 49).	How do participants enroll others into helping and supporting them? What do they need to do to reciprocate? How do caregivers integrate supportive work into their daily lives (do they ration care, time, emotional support etc).	Who are the people who help you most in managing your health? In what ways do they help you? How do you get the support you need from them? What do you need to do to reciprocate?<br><br>*For the carer:*<br>How do you cope with your care-giving role? How do you feel about it?
	**Competence**	Patients and caregivers demonstrate competence when they are able to exert control over disease progression (36), effectively participate in self-management (48), understand multimorbidities (10, 17), and manage polypharmacy (28).	What do participants need to do to demonstrate that they are competent and compliant users of health and healthcare services? What are the tasks that come from participating in healthcare?	What tasks do you need to do to manage/control your two health conditions (at home/work, re health services)? How did having a second condition add to your tasks? What tasks are you managing well/not so well, and why? What could help you manage certain tasks better? What makes it hard to manage all these tasks?<br><br>*For the carer:*<br>What are your tasks as the main carer? What makes it hard to manage all these tasks?<br>What could help you in your role?

	**Help-seeking**	Patient and caregiver help seeking is governed by interactions between expectations of clinical interventions (9), and help-seeking (10, 28, 33, 37). Help-seeking is framed mainly thorugh rational responses to emergency situations (8), and patients and caregivers valued the security and safety of hospital care (8, 37).	What help-seeking activities are routinely experienced by participants? What help-seeking activities are experienced by participants in emergencies? How (and by who) is an emergency defined? What processes lead to in-patient care as a solution to emergencies, and who interacts with healthcare providers to organize and manage these processes?	Do you feel able to ask for help and get everything you need? Do you know where to get help from?<br>What do you do when you need urgent help with your condition? Who helps you make decisions in a health crisis?<br>Have you ever experienced a health emergency? If so can you tell me how you went about getting help and making decisions about what do to?<br>Has having HIV/T2D/Dep made it easier or more difficult in getting the healthcare you need?<br><br>*For the carer:*<br>Have you ever had to help the pt get help in a health crisis? If yes, what did you do?
	**Technological support**	Technological interventions as life sustaining (8, 12); as mastery (29, 49, 50); as burdens (24, 30, 47); as intrusions (21, 49); Risks of treatment failure/complications (49, 50).	What kinds of hardware are available to participants? How to they use these to manage their health and healthcare problems?	What do you know about any technologies that can help you manage your condition? Do you use any? If so how do they help? If not, why not?<br>What aids would you like to have to help you?<br><br>*For the carer:*<br>Do you know of any technologies that could help you with your caring tasks? If yes, please describe.

	**Situated decision-making**	Patient and caregiver decision-making about help-seeking and service utilization was framed by the degree of awareness and uncertainy about prognosis (13, 19, 26, 27, 38); Difficulty and conflict over decisions (12, 51), could be ameloriated using tools and techniques to increase patient control (19, 21, 34, 48).	How do participants experience pathophysiological deterioration? How do they (and others) manage uncertainty over treatment regimens, outcomes of treatment and care processes, and negotiations about access to care? What techniques do they use to control their experiences of health and healthcare?	How confident are you about managing all the tasks and decisions involved in managing your health condition and getting the care you need? How much control do you feel you have over how to manage your condition?<br><br>*Prompts:* How easy or difficult do you find all of these activities? How do you feel when things feel out of control (eg. When you have symptoms and don't feel well)? And what helps make you feel more in control again?<br><br>What about in the future?<br>*Prompts:* What are your expectations of your health in the future? What do you expect from the health services in the future? How confident do you feel about the quality of the healthcare you will receive in the future?<br><br>*For the carer:*<br>How do you see your role in the future? What are your expectations for how things are going to be going forward?

REFERENCES

1. Strachan PH, Currie K, Harkness K, Spaling M, Clark AM. Context matters in heart failure self-care: a qualitative systematic review. J Card Fail. 2014;20(6):448-55.
2. Langer S, Chew-Graham C, Hunter C, Guthrie EA, Salmon P. Why do patients with long-term conditions use unscheduled care? A qualitative literature review. Health & Social Care in the Community. 2013;21(4):339-51.
3. Thomas JR, Clark AM. Women with heart failure are at high psychosocial risk: A systematic review of how sex and gender influence heart failure self-care. Cardiology Research and Practice. 2011;1(1).
4. Rolls TP, Young LE. Disrupting the Biomedical Discourse: Older Women's Lived Experiences with Heart Failure: A Feminist Review of the Literature. Canadian Journal of Cardiovascular Nursing. 2012;22(1):18-25.
5. Tong A, Jesudason S, Craig JC, Winkelmayer WC. Perspectives on pregnancy in women with chronic kidney disease: systematic review of qualitative studies. Nephrol Dial Transplant. 2015;30(4):652-61.
6. Yu DS, Lee DT, Kwong AN, Thompson DR, Woo J. Living with chronic heart failure: a review of qualitative studies of older people. J Adv Nurs. 2008;61(5):474-83.
7. Hopp FP, Thornton N, Martin L. The lived experience of heart failure at the end of life: a systematic literature review. Health Soc Work. 2010;35(2):109-17.
8. de Sousa Pinto JM, Martin-Nogueras AM, Morano MT, Macedo TE, Arenillas JI, Troosters T. Chronic obstructive pulmonary disease patients' experience with pulmonary rehabilitation: a systematic review of qualitative research. Chronic Respiratory Disease. 2013;10(3):141-57.
9. Oishi A, Murtagh FE. The challenges of uncertainty and interprofessional collaboration in palliative care for non-cancer patients in the community: a systematic review of views from patients, carers and health-care professionals. Palliat Med. 2014;28(9):1081-98.
10. Thorpe O, Johnston K, Kumar S. Barriers and enablers to physical activity participation in patients with COPD: a systematic review. J Mol Signal. 2012;32(6):359-69.
11. McEntee ML, Cuomo LR, Dennison CR. Patient-, provider-, and system-level barriers to heart failure care. J Cardiovasc Nurs. 2009;24(4):290-8.
12. Dev S, Abernethy AP, Rogers JG, O'Connor CM. Preferences of people with advanced heart failure-a structured narrative literature review to inform decision making in the palliative care setting. Am Heart J. 2012;164(3):313-9.e5.
13. Low J, Pattenden J, Candy B, Beattie JM, Jones L. Palliative care in advanced heart failure: an international review of the perspectives of recipients and health professionals on care provision. J Card Fail. 2011;17(3):231-52.
14. Jani B, Blane D, Browne S, Montori V, May C, Shippee N, et al. Identifying treatment burden as an important concept for end of life care in those with advanced heart failure. Curr. 2013;7(1):3-7.
15. Jeon YH, Kraus SG, Jowsey T, Glasgow NJ. The experience of living with chronic heart failure: a narrative review of qualitative studies. BMC Health Services Research. 2010;10:77.
16. Kang X, Li Z, Nolan MT. Informal caregivers' experiences of caring for patients with chronic heart failure: systematic review and metasynthesis of qualitative studies. J Cardiovasc Nurs. 2011;26(5):386-94.
17. Dickson VV, Buck H, Riegel B. A qualitative meta-analysis of heart failure self-care practices among individuals with multiple comorbid conditions. J Card Fail. 2011;17(5):413-9.

18. Siabani S, Leeder SR, Davidson PM. Barriers and facilitators to self-care in chronic heart failure: a meta-synthesis of qualitative studies. Springerplus. 2013;2:320.
19. Moustakas J, Bennett PN, Nicholson J, Tranter S. The needs of older people with advanced chronic kidney disease choosing supportive care: a review. Renal Society of Australasia Journal. 2012;8(2):70-5.
20. Morton RL, Tong A, Howard K, Snelling P, Webster AC. The views of patients and carers in treatment decision making for chronic kidney disease: systematic review and thematic synthesis of qualitative studies. BMJ. 2010;340:c112.
21. Harwood L, Clark AM. Understanding pre-dialysis modality decision-making: A meta-synthesis of qualitative studies. International Journal of Nursing Studies. 2013;50(1):109-20.
22. Falk H, Ekman I, Anderson R, Fu M, Granger B. Older patients' experiences of heart failure-an integrative literature review. J Nurs Scholarsh. 2013;45(3):247-55.
23. Giacomini M, DeJean D, Simeonov D, Smith A. Experiences of living and dying with COPD: a systematic review and synthesis of the qualitative empirical literature. Ont Health Technol Assess Ser. 2012;12(13):1-47.
24. Sookhoo D, Pellowe C, Derham C. The experiences of heart failure patients following their participation in self-management patient education programmes: A systematic review. JBI Database of Systematic Reviews and Implementation Reports. 2013;11(2):236-80.
25. Clark AM, Spaling M, Harkness K, Spiers J, Strachan PH, Thompson DR, et al. Determinants of effective heart failure self-care: a systematic review of patients' and caregivers' perceptions. Heart. 2014;100(9):716-21.
26. Disler RT, Green A, Luckett T, Newton PJ, Inglis S, Currow DC, et al. Experience of advanced chronic obstructive pulmonary disease: metasynthesis of qualitative research. J Pain Symptom Manage. 2014;48(6):1182-99.
27. Welstand J, Carson A, Rutherford P. Living with heart failure: An integrative review. International Journal of Nursing Studies. 2009;46(10):1374-85.
28. Gysels M, Bausewein C, Higginson IJ. Experiences of breathlessness: a systematic review of the qualitative literature. Palliat Support Care. 2007;5(3):281-302.
29. Tong A, Lesmana B, Johnson DW, Wong G, Campbell D, Craig JC. The perspectives of adults living with peritoneal dialysis: thematic synthesis of qualitative studies. Am J Kidney Dis. 2013;61(6):873-88.
30. Wadd K, King L, Bennett P, Grant J. Being a parent on dialysis: a literature review. J. 2011;37(4):208-15.
31. Dekker RL. Patient perspectives about depressive symptoms in heart failure: a review of the qualitative literature. J Cardiovasc Nurs. 2014;29(1):E9-15.
32. Tong A, Cheung KL, Nair SS, Kurella Tamura M, Craig JC, Winkelmayer WC. Thematic synthesis of qualitative studies on patient and caregiver perspectives on end-of-life care in CKD. Am J Kidney Dis. 2014;63(6):913-27.
33. Clark AM, Savard LA, Spaling MA, Heath S, Duncan AS, Spiers JA. Understanding help-seeking decisions in people with heart failure: A qualitative systematic review. International Journal of Nursing Studies. 2012;49(12):1582-97.
34. Makaroff KL. Experiences of kidney failure: a qualitative meta-synthesis. Nephrol Nurs J. 2012;39(1):21-9, 80; quiz 30.
35. Tierney S, Mamas M, Skelton D, Woods S, Rutter MK, Gibson M, et al. What can we learn from patients with heart failure about exercise adherence? A systematic review of qualitative papers. Health Psychol. 2011;30(4):401-10.
36. Harrison SL, Apps L, Singh SJ, Steiner MC, Morgan MD, Robertson N. 'Consumed by breathing' - a critical interpretive meta-synthesis of the qualitative literature. Chronic Illness. 2014;10(1):31-49.

37. Keating A, Lee A, Holland AE. What prevents people with chronic obstructive pulmonary disease from attending pulmonary rehabilitation? A systematic review. Chronic Respiratory Disease. 2011;8(2):89-99.
38. Barclay S, Momen N, Case-Upton S, Kuhn I, Smith E. End-of-life care conversations with heart failure patients: a systematic literature review and narrative synthesis. Br J Gen Pract. 2011;61(582):e49-62.
39. Procter E. Collaboration between the specialties in provision of end-of-life care for all in the UK: reality or utopia? Int J Palliat Nurs. 2012;18(7):339-47.
40. Bayhakki, Hatthakit U. Lived experiences of patients on hemodialysis: a meta-synthesis. Nephrol Nurs J. 2012;39(4):295-304; quiz 5.
41. Disler RT, Gallagher RD, Davidson PM. Factors influencing self-management in chronic obstructive pulmonary disease: an integrative review. International Journal of Nursing Studies. 2012;49(2):230-42.
42. Palmer SC, Hanson CS, Craig JC, Strippoli GF, Ruospo M, Campbell K, et al. Dietary and Fluid Restrictions in CKD: A Thematic Synthesis of Patient Views From Qualitative Studies. Am J Kidney Dis. 2015;65(4):559-73.
43. Wingham J, Harding G, Britten N, Dalal H. Heart failure patients' attitudes, beliefs, expectations and experiences of self-management strategies: a qualitative synthesis. Chronic Illness. 2014;10(2):135-54.
44. Harkness K, Spaling MA, Currie K, Strachan PH, Clark AM. A systematic review of patient heart failure self-care strategies. J Cardiovasc Nurs. 2015;30(2):121-35.
45. Buck HG, Harkness K, Wion R, Carroll SL, Cosman T, Kaasalainen S, et al. Caregivers' contributions to heart failure self-care: A systematic review. European Journal of Cardiovascular Nursing. 2015;14(1):79-89.
46. Molloy GJ, Johnston DW, Witham MD. Family caregiving and congestive heart failure. Review and analysis. Eur J Heart Fail. 2005;7(4):592-603.
47. Cullen DL, Stiffler D. Long-term oxygen therapy: Review from the patientsg perspective. Chronic Respiratory Disease. 2009;6(3):141-7.
48. Low J, Smith G, Burns A, Jones L. The impact of end-stage kidney disease (ESKD) on close persons: A literature review. NDT Plus. 2008;1(2):67-79.
49. Walker RC, Hanson CS, Palmer SC, Howard K, Morton RL, Marshall MR, et al. Patient and caregiver perspectives on home hemodialysis: a systematic review. Am J Kidney Dis. 2015;65(3):451-63.
50. Casey JR, Hanson CS, Winkelmayer WC, Craig JC, Palmer S, Strippoli GF, et al. Patients' perspectives on hemodialysis vascular access: a systematic review of qualitative studies. Am J Kidney Dis. 2014;64(6):937-53.
51. Luckett T, Sellars M, Tieman J, Pollock CA, Silvester W, Butow PN, et al. Advance care planning for adults with CKD: a systematic integrative review. Am J Kidney Dis. 2014;63(5):761-70.

<u>Interview Schedule EXTRA – DRAFT 2.0</u>

4. Structural, spatial, and systemic disadvantages are important factors that inhibit active engagement with formal healthcare and self-management.

a. Socio-economic status

Main question: How do you think your age/gender/income affects your health (physical; mental) and your experience of health care?

Prompts for patients:

- What are the costs associated with caring for your health?
- Are there things that you need to take care of your health that you cannot afford (medicines or remedies other than from the clinic; food; aids; services)?
- How does your age affect your access to health services, or the way HCPs treat you or talk to you?
- Is there anything that you do to try and overcome such difficulties?

Prompts for carers:

- What are your thought about these issues?
- Is there anything you would like to add which have not been addressed?

b. Spatial location

Main question: What are some of the difficulties you experience in getting to health services (clinic; hospital)?

Prompts for patients:

- How do you think time, work and family commitments and transport impacts your ability to get care (go to the clinic, get medication, etc)?
- How do you think the area in which you live affects your ability to manage your health well (crime; diet; physical activity; availability of community resources)?
- Are you able to afford the medication, aids, and services you need? (if yes …)
- What do you need to do to overcome the challenges you experience?
- How does your age affect your access to health services, or the way HCPs treat you or talk to you?

Prompts for the carer:

- What are the difficulties you, as the carer, experience in helping the patient access services?
- What are your thoughts/feelings about these issues?

5. **System quality**

Main question: What is your experience of the health services you use? What is good/not so good? What would you like to see improved/changed?

Prompts for patients

- How would you describe your interactions with healthcare services and healthcare providers?
- What work would you say requires you to engage with health services and healthcare providers?
- Are there specific system or professional problems that you need to overcome? What do these look like?
- How easy is it once you are there, to know what you need to do, where to go to get the assistance you need from clinic staff?

HC environment

- Can you tell me more about the quality of the facilities (cleanliness; security; comfort; toilets)?

Organisational

- What do you think of the way the health services are organized (opening and closing times; waiting times; queue management; co-ordination and continuity of care; role of different *HCPs;* location of pharmacy; referral; pt records)?

Clinical staff

- What is your experience of the drs and nurses (perceived expertise/clinical skills; professionalism; attitude to pts - way they treat and talk to pts)?

Other staff (security; admin)

- How professional and efficient are other staff at the clinic? How would you describe the way staff engage with the services and patients?

6. **Patients and caregivers experience multiple affective, cognitive, and interactional disadvantages as they seek to participate in encounters with clinicians, decisions about their formal healthcare and self-management processes. Cognitive advantage**

Prompts for patients

- Can you tell me what information you want to find out about your health problems?
- Who do you need to engage with to do this? What work does require you to do?
- When you receive the information, how do you relate it to your circumstance?
- What do participants do to understand and prepare for symptoms and their exacerbation, what work is involved in this, what support is available?
- What do you understand about your conditions?
- How have you gained info/knowledge about your different conditions and how to take care of your health?
- What would you say are your best sources of information?
- Do you trust this information?
- Do you get different information from different sources that you find confusing?
- How do you make this information relevance to your personal circumstances?
- What do you think you know enough about? And what would you like to know more about?
- When you experience symptoms that concern you and you need to understand more about them, where do you go to get information and support?

Prompts for the carer:

- How have you learnt about these conditions of the patient? How do help the patient manage the condition?

- How well informed do you think you are about the condition?
- Have you had access to any resources which specifically address your needs as a carer? If so, please describe. [what are the needs of the patient you care for?]

7. **Affective state**

Main question: How has having more than one health condition or illness affected you emotionally?

Prompts for patients

- How do you experience and manage issues connected with stigma, self-esteem, and social functioning?
- What problems would you say you face?
- What do you need to do to achieve this? (to address the problems)
- How did getting a diagnosis of another chronic or long-term condition/s affect you emotionally?
- Which one would you say has had the greatest impact on you?
- Who can you depend on for emotional support?
- Has having more than one chronic condition affected your personal relationships in any way?
- Has it affected the way you see yourself or think about yourself?

Prompts for the carer:

- Do you have anything you would like to add from your perspective?
- How have you coped emotionally as the patients main carer?

8. **Interaction quality**

Main question: Patients and caregivers value resilience, functional performance and social support that make a practical contribution to formal healthcare and self-management.

Prompts for the patient:

- How would you describe your relationship with HCPs (drs and nurses)?
- How would you say they help you?
- How do you feel when interacting with them?
- How are you in the interaction with them?
- How do you feel about decision making in relation to how to manage your health?
- What do you think would improve the quality of your interaction with your HCPs?
- Does having more than one chronic condition change anything in your relationship with HCPs?

For the carer:

- What is your experience of interacting with the HCP when you accompany the patient?

9. **Adaptation to disruption**

Main question: What do participants do to adapt to their health and healthcare problems?

Prompts for the patient:

- How do you experience physiological and psychological effects and limitations?
- What self-management strategies do you use (if any)?
- How do you 'smooth out' the disruptive effects of health and healthcare problems?
- How has having more than one chronic condition changed your life (physical & psychological effects; limitations on way of life, work, and routines)
- How has it impacted your relationships, family life?
- How was it to have a second or third or more chronic conditions to cope with?
- How have you coped with these changes?
- What has helped you adapt to living with two or more chronic conditions?
- How easy or difficult has it been to accept our situation?
- How have you overcome some of the difficulties you have faced?
- How do you manage work in your situation (time off for appointments and illness; attitude of co-workers and boss; limitations on ambitions?)

For the carer:

- How has your caring role affected or changed your life (eg: limited your activities; changed your routine and relationships)?
- How have you adapted to these changes?

10. **Caregiver support**

Main question: Who is helping/supporting patients manage their healthcare? Understanding the caregivers

Prompts for the patient:

- How do you enroll/get others into helping and supporting you?
- What do you need to do to reciprocate?
- How do caregivers integrate supportive work into their daily lives (do they ration care, time, emotional support etc)?
- Who are the people who help you most in managing your health?
- In what ways do they help you?
- How do you get the support you need from them?
- What do you need to do to reciprocate?

For the carer:

- How do you cope with your care-giving role? How do you feel about it?

11. Competence

Main question: What do participants need to do to demonstrate that they are competent and compliant users of health and healthcare services? What are the tasks that come from participating in healthcare?

Prompts for patients:

- What tasks do you need to do to manage/control your different health conditions (at home/work, re health services)?
- How did having a diagnosis of a further condition/s add to your tasks?
- What tasks are you managing well/not so well, and why? What could help you manage certain tasks better?
- What makes it hard to manage all these tasks for you?

For the carer:

- What are your tasks as the main carer?
- What makes it hard to manage all these tasks?
- What could help you in your role?

12. Help seeking

Main question: What help-seeking activities are routinely experienced by participants?

Prompts for the patient:

- What help-seeking activities are experienced by participants in emergencies?
- How (and by who) is an emergency defined?
- What processes lead to in-patient care as a solution to emergencies, and who interacts with healthcare providers to organize and manage these processes?
- Do you feel able to ask for help and get everything you need? Do you know where to get help from?
- What do you do when you need urgent help with your condition? Who helps you make decisions in a health crisis?
- Have you ever experienced a health emergency? If so, can you tell me how you went about getting help and making decisions about what do to?
- Has having multiple chronic conditions made it easier or more difficult in getting the healthcare you need?

For the carer:

- Have you ever had to help the patient get help in a health crisis? If yes, what did you do?

13. Technological support

Main question: What kinds of hardware are available to participants? How to they use these to manage their health and healthcare problems?

Prompts for patients:

- What do you know about any technologies, (eg something on your cell phone that measures exercise or reminds you to take medication; or a glucose meter or blood pressure machine) that can help you manage your condition?
- Do you use any? If so, how do they help? If not, why not?
- What aids would you like to have to help you?

For the carer:

- Do you know of any technologies that could help you with your caring tasks? If yes, please describe.
- If not, would these look for you?

14. Situated decision-making

Main question:

Prompts for the patient

- How have you experienced pathophysiological deterioration?
- How do you (and potentially other carers) manage uncertainty over treatment regimens/routines?
- What have been the outcomes of treatment and care processes, and negotiations about access to care?
- What techniques do you use to control their experiences of health and healthcare?
- How confident are you about managing all the tasks and decisions involved in managing your health condition and getting the care you need?
- How much control do you feel you have over how to manage your condition?

Prompts for patients:

- How easy or difficult do you find all these activities?
- How do you feel when things feel out of control (eg. When you have symptoms and do not feel well)?
- And what helps make you feel more in control again?
- What are your expectations of your health in the future?
- What do you expect from the health services in the future?
- How confident do you feel about the quality of the healthcare you will receive in the future?

For the carer:

- How do you see your role in the future? What are your expectations for how things are going to be going forward?

1. Affective state

Main Question: *How has having more than one health condition or illness affected you emotionally?*	

Extra prompts for patients

• When you got the diagnosis of your first & second (or third) condition, how did this make you feel? And can tell you me a bit more about when you were diagnosed with your conditions (medical history)	
• What condition do you find harder to live with, emotionally?	
• How has having multiple chronic conditions changed your life? (physically, emotionally, economically) Has it changed you as a person or your relationships? Has it changed the way you live your life, limited you in any way? If yes, please explain how….	
• Has having multiple conditions changed the way you see yourself or think about yourself?	
• Have you ever experienced negative attitudes (stigma) from others? And how did you react to this?	
• How do you feel about having these conditions (positive/negative emotions)? Do you ever feel depressed when you are thinking about your conditions?	

2. Adaptation to disruption

Main question: *How has having more chronic conditions changed your life (physical & psychological effects; limitations on way of life, work, and routines; impact on relationships; family life)?*	

Extra prompts for the patient:

• How is it to live with two or more chronic conditions physically and emotionally?? And how are you coping with the changes to your life?	
• How easy or difficult has it been to accept your situation? How have you overcome some of the difficulties you have faced?	
• What has helped you adapt to living with two or more chronic conditions?	
• How has having more than one chronic condition changed your life (physical & psychological effects; limitations on way of life, work and routines)	
• How do you manage work in your situation (time off for appointments and illness; attitude of co-workers and boss; limitations on ambitions?)	
• What do you do to cope with your conditions? Are there particular	

strategies you use to stay healthy? (eg. a strict sleeping routine, having a pill box, counselling, joining a support club etc).	

Extra prompts for the carers

• How has your caring role changed your life (eg: limited your freedom, changed your routine and relationships)?	
• How have you adapted to these changes?	

3. Socio-economic status

Main Question: *How do you think your age/gender/income affects your health (physical; mental) and your experience of health care?*	

Extra prompts for patients

• How do you think your age or being male or female affects your health and the care you get from the clinic? (your health can be physical or psychological/emotional).	
• And how do you think how much money you have affects your health and your (access to) care? For example, is it easy to pay for medication, special food, or aids that you need for your conditions?	
• Are there things that you need to take care of your health that you cannot afford? If yes, can you tell me what these are?	

Prompts for carers

• What are your thoughts about the money that the patient needs to spend to keep healthy? health needs? (consider time, diet, transport etc)<br>• Do you think that age or gender influence the care the patient gets at the clinic/hospital?	

4. Spatial location of services

Main Question: *What are some of difficulties you experience in getting to health services (clinic; hospital)?*	

Extra prompts for patients

• How do you think time, work and family commitments and transport impacts your ability to get care (go to the clinic, get medication, etc)?	
• How do you think the area in which you live influences your health and getting health and/or social cOare (crime; diet; physical activity; availability of community resources)? Can you also tell me how you are getting to the facilities?	
• How do you deal with these problems (if present)?	

Extra prompts for the carers

• What are the difficulties you, as the carer, experience in helping the patient get the health and/or social care they need from the clinic?	
• What are your thoughts/feelings about these issues?	

5. System quality

Main question: *What is your experience of the health services you use? What is good/not so good about them? What would you like to see improved/changed?*	

Extra prompts for patients

• What do you think of the way the health services are organized?<br>o Opening times; waiting times; queue management; the frequency of visits; role of different health workers; location of pharmacy; referral to other doctors or the hospital; patient records<br>o The condition of facilities such as bathrooms, benches etc<br>o Do you feel safe/secure when visiting in the clinic?<br>o How do you think health services will change in the future (eg. Provision of health care, extra support, monetary support)?	
• Once you are at the clinic, how easy is it to find out where you need to go, or which doctor or nurse you need to see? What difficulties do you experience when in the clinic?	

Extra prompts for carers

• What has been your experience of the quality of health care when you go with the patient to the clinic?<br>o General services, admin, staff, staff attitude, waiting time etc.<br>o How do you think health services will change in the future (eg. Provision of health care, extra support, monetary support)?	

6. Interaction quality (Interaction with health workers)

Main Question: *How would you describe your relationship with health workers?*	

Extra prompts for patients

• Can you describe the relationship you have with your health worker(s)? What do you like about them, what can they do better or improve?	
• How do you, as a patient, feel when you are interacting with them?	
• How do your health workers help you manage your conditions?	
• Has having more than one chronic condition changed anything in your	

relationship with health workers?	
• How do you feel about decision making in relation to how to manage your health?	
• What do you think would improve the quality of your interaction with your HCPs?	
• Is there anything else you would like to share about your relations with your health workers?	

Extra prompts for the carers

• Can you tell me more about your interaction with health worker(s) when you come with the patient to the clinic?	
• Is there anything that they (the health workers) could improve to help you or the patient?	

7. Technological support

Main Question: *How do you as a patient use technology (cell phones, aids, computers) to help you manage your conditions?*	

Extra prompts for patients

• What do you know about technologies that can help you manage your conditions? Do you use any? Such as SMS reminders, apps, glucometer etc?	
• Do you have any cellphone apps that remind you to take your medication?	
• Do you have a glucose meter or blood pressure machine to help you? And yes, how often do you use it?	
• Are you comfortable using the technologies available to you? Why do you find it easy or difficult?	
• How does the technology you mentioned help manage your conditions?	
• What other type or technology (such as app, SMSes etc) would you like to use? And how do you think this will help you?	

Extra prompts for the carer

• Do you know of any technologies that could help you with your caring tasks? If yes, please describe.	
• Do you have access to any of these technologies? And how would they help you?	

8. Cognitive advantage (health information)

Main Question: *What do you understand about your conditions? How have you gained info/knowledge about your different conditions and how to take*	

care of your health?	

Extra prompts for patients

• What kind of (health) information do you get about your conditions? For example through pamphlets, doctor's advice, talks at health facilities, radio, TV shows, internet?	
• Is this information relevant to you? Do you trust it? And is it useful? And is it easy to read? And is the information consistent or do you find it confusing?	
• What would you say are your best sources of information?	
• Do you have enough information? And what are you missing?	
• What do you think you know enough about? And what would you like to know more about?	
• If you have a particular symptom that is scary/concerning, where do you go to get extra information and support?	

Extra prompts for the carers

• How did you learn more about the condition of the patient? Did you get pamphlets, speak to health worker, get information from radio/TV/internet? Was it useful?	
• Did you receive any information specifically for your role as a carer? If yes, what kind of information is that?	
• How well informed do you think you are about the condition?	

9. Caregiver support

Main Question: *Who is helping/supporting you to manage your own healthcare?*	

Extra prompts for the patients

• Can you please tell me more about the person who came with you to the interview. Is he/she your main caregiver?	
• How do they help you in your daily routines? And what do you appreciate the most? Can you tell me more about your relationship?	
• Do you do something to thank your carer for their service? And if yes, what do you do?	
• How do you think that your carers combine supporting you with other tasks they need to do? For instance, do you think they have enough time to care for you, how do they cope emotionally and is the work hard?	
• Is there anyone else in your network who assists you?	

Extra prompts for the carer

• How do you cope with your care-giving role? How do you feel about it?	
• Can you tell us more about your experience of care giving? Has it become harder/easier over time? And why?	

10. Help-seeking

**Main Question:** *Have you ever had to get help for a health emergency? And how do you seek help when you have concerns about your health conditions? Can you tell me more?*	

Extra prompts for the patient

• If you need help with managing one or more of your conditions, do you feel you can ask for help? Where do you get the help from? For example, do you speak to a counsellor, family member or neighbour?	
• What do you do when you need urgent help with your condition? Who helps you make decisions in a health crisis (emergencies) (eg. Who speaks to the doctor/nurse, makes sure you get to the clinic)	

Extra prompts for the carer

• Have you ever had to help the patient get help in a health crisis/emergency? If yes, what did you do? And how did it make you feel?	

11. Situated decision-making

Main Question: *Do you think that you (as a patient) manage your conditions well? And can you explain why/why not?*	

Extra prompts for the patient

• How easy or difficult do you find all the things you have to do to stay healthy (eg. Taking medications, diet, activities)? Is it stressful? And why? Which condition is the hardest to manage?	
• How do you feel when things are out of control (eg. When you have symptoms and do not feel well)?	
• How much control do you feel you have over your conditions? And can you explain why?	
• And what helps to make you feel more in control of your health? Do you have particular strategies?	
• What could make it easier to manage your conditions in future?	
• Do you think the quality of health services will improve in the future? And what do you thinks need to be changed to make the services better?	

Extra prompts for the carer

• How do you see your role as a carer in the future?	
• How do you think health services will change in the future (eg. Provision of health care, extra support, monetary support)?	

12. Competence

Main Question: *As a patient, how do you show the nurses/doctors or your carer that you are trying to stay healthy and do what you are supposed to do?*	

Extra prompts for the patient

• What do you need to do to stay healthy? How often do you need to take medication, what foods do you need to eat, how often do you go for check-ups? What else?	
• What tasks are you managing well/not so well, and why? What could help you manage certain tasks better?	
• What tasks are easy for you to stay healthy? And what is difficult? And why are they hard/easy? What could help you manage certain tasks better?	

Extra prompts for the carer

• What are your tasks as the main carer?	
• What makes it hard to manage all these tasks? And what is easy?	
• What could help you in your role?	

13. Covid-19 Questions

Main Question: *How has COVID-19 impacted how you take care of yourself and your health?*	

Extra prompts for patient

• Has COVID-19 made it easier or harder to stay healthy? And why? For example, has it been more difficult to get food, have you lost income, do you feel less safe, is it more difficult to get around?	
• What precautions have you been taking to be safe when seeking health care? Have these been changes been easy or difficult?	

Extra prompts for the carer

• How has COVID-19 changed your role as a carer?	
• What precautions have you been taking to ensure the safety of yourself and patient?	
• Has COVID-19 made it easier or harder for you to assist the patient? And why?	
